# Case Report: Laparoscopic resection of a tumor in the right posterior lobe of the liver in a patient with situs inversus totalis

**DOI:** 10.3389/fonc.2025.1513584

**Published:** 2025-03-25

**Authors:** Bin Yang, Xin Luo, Genjun Mao

**Affiliations:** Department of Hepatopancreatobiliary Surgery, Affiliated Jinhua Hospital, Zhejiang University School of Medicine, Jinhua Municipal Central Hospital, Jinhua, Zhejiang, China

**Keywords:** complication, hepatectomy, liver tumor, minimally invasive laparoscopy, situs inversus totalis, case report

## Abstract

**Background:**

Liver cancer poses a major health burden worldwide. Therefore, surgical resection is an important treatment option for patients with liver cancer. In situs inversus totalis (SIT), a rare genetic condition, organs such as the heart, liver, spleen, and stomach are unpaired in the chest and abdominal cavity and their positions are opposite their usual positions.

**Case presentation:**

We report a case of SIT with a tumor in the right posterior lobe of the liver in a 70-year-old man. This patient was successfully treated with laparoscopic resection.

**Conclusions:**

This case emphasizes that laparoscopic hepatectomy, comprising comprehensive preoperative preparation and fine intraoperative cooperation, is safe and feasible in patients with SIT.

## 1 Introduction

Liver cancer is the sixth most common cancer and has the third highest mortality rate worldwide (1). In 2020, more than 830,000 individuals died of liver cancer. Furthermore, the incidence of liver cancer in China increased from 258,000 cases in 1990 to 515,900 in 2017 (2). Hepatitis B virus is considered the main cause of liver cancer. Although the incidence of liver cancer has decreased with the popularization of the hepatitis B vaccine, it remains a major public health concern.

Situs inversus totalis (SIT) is defined as the mirror image distribution of the viscera and normal anatomical structures (3). Hong et al. observed a case of SIT during laparoscopic right hemihepatectomy in South Korea, but very few relevant cases have been reported in China (4). Patients with SIT do not have a pathophysiology different from that of individuals without SIT; however, when visceral diseases develop, these congenital anatomical abnormalities can affect diagnosis and surgical procedures.

In 2019, despite technical challenges, we successfully performed laparoscopic resection of segments VI and VIII of the right posterior lobe of the liver in a patient with SITS. Information regarding this case could be helpful in managing other patients with SIT and liver cancer.

The authors are accountable for all aspects of the work in ensuring that questions related to the accuracy or integrity of any part of the work are appropriately investigated and resolved. Each patient provided written informed consent to participate in the study. The research was approved by the Ethics Committee of Jinhua Municipal Central Hospital. The study was conducted in accordance with the Declaration of Helsinki (revised in 2013).

## 2 Case presentation

A 70-year-old man underwent routine health check-up at the community health center 3 days before and was admitted after the discovery of a liver tumor. Based on the patient’s CT and MRI results, the patient was diagnosed with hepatocellular cancer (HCC). The patient reported no obvious symptoms of discomfort, history of unusual illnesses, alcohol consumption, history of surgery, or significant weight loss before admission.

**Figure 1:**
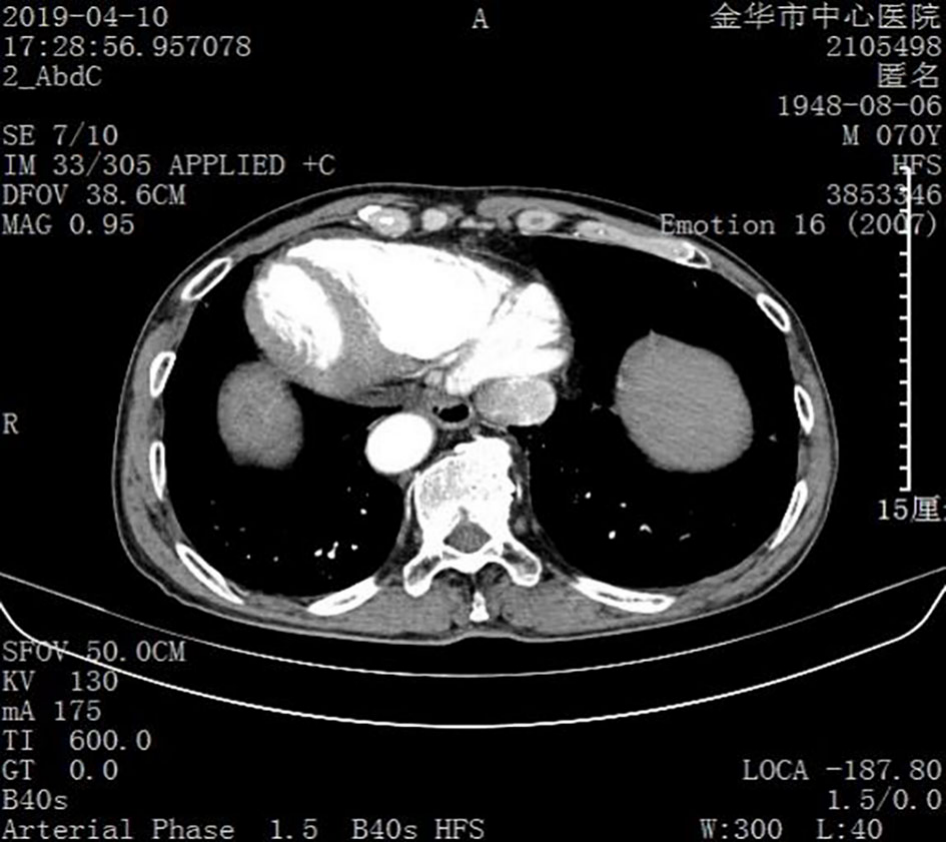
Image obtained using computed tomography. The organs are inverted.

**Figure 2:**
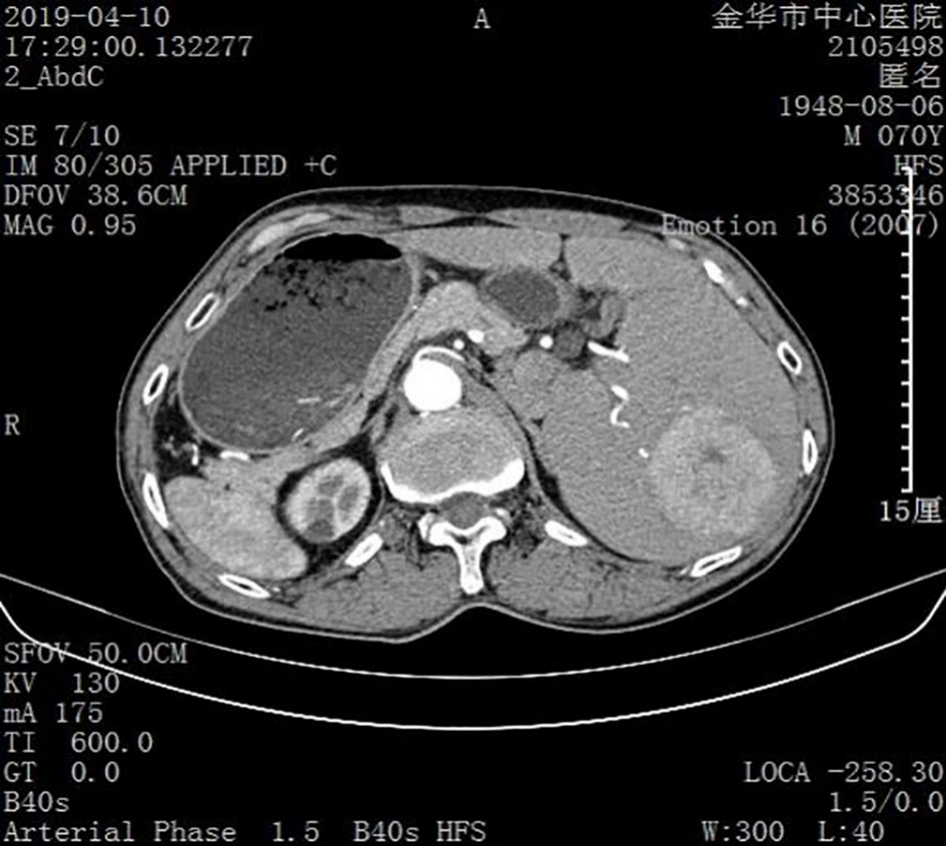
Image obtained using enhanced computed tomography. The liver and tumor are located in the left abdominal cavity.

SIT was diagnosed based on the results of an imaging examination. Ultrasound examination suggested visceral inversion and a heterogeneous hypoechoic mass (approximately 6.0 cm × 5.0 cm) in the right liver. Enhanced computed tomography and magnetic resonance imaging (MRI) examinations indicated that the liver was located in the left abdominal cavity and that the tumor was located in segments VI and VIII of the right posterior lobe of the liver (**Figures 1**, **2**).

The patient had a body mass index of 21.1 kg/m^2^. A routine blood examination yielded the following results: white blood cell count, 5.4×10^9^/L; hemoglobin level, 136 g/L; platelet count, 228×10^9^/L; alpha-fetoprotein level, 4.03 ng/mL; albumin level, 42.8 g/L; total bilirubin level, 25.1 umol/L; and thrombin time, 12.4 s. The hepatitis B surface antigen, hepatitis B e antibody, and hepatitis B core antibody results were positive, and quantification of the hepatitis B virus indicated a level below the detection limit. The Child-Pugh liver function grade was class A, and the indocyanine green retention rate at 15 min was 4.6%. Based on the evaluation of the patient’s Child-Pugh score and indocyanine green retention test result, we concluded that the patient’s liver function was within normal limits.

Based on the patient’s imaging data, the tumor was classified as early-stage liver cancer characterized by a single lesion without distant metastasis. Following multidisciplinary team discussion and consultation with the patient’s family members, it was determined that complete surgical resection represented the optimal curative approach for early-stage liver cancer. Although liver cancer has a certain sensitivity to radiotherapy and chemotherapy, early-stage liver cancer may achieve the best benefits after surgical treatment.

**Figure 3:**
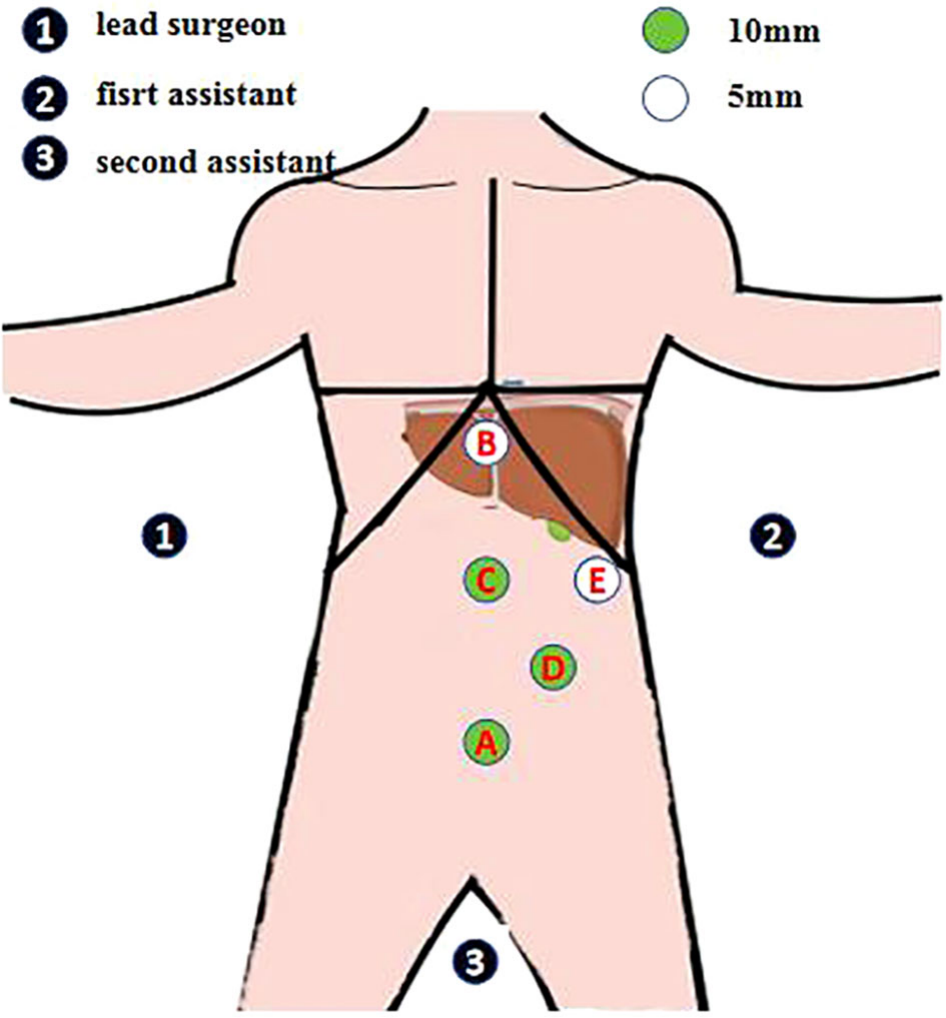
Abdominal five-hole method. A 10-mm trocar is placed at the lower edge of the umbilical foramen as hole A A 5-mm trocar is placed 1.0 cm below the xiphoid process as hole B A 10-mm trocar is placed at the midpoint of the line connecting holes B and A, thus creating hole C A 10-mm trocar is horizontally placed 3.0 cm above the left clavicle midline umbilicus as hole D A 5-mm trocar is placed below the left axillary anterior rib as hole E The lead surgeon uses holes B and C during surgery; however, hole C is the main operating hole. The first assistant uses holes D and E.

Under general anesthesia, the patient was positioned with the legs separated. The lead surgeon stood on the right side of the patient and the first assistant stood on the left side of the patient. During surgery, the operating table was adjusted to tilt the patient’s body to the right by approximately 30°, the patient’s head remained high, and the feet remained low. Laparoscopic hepatectomy was performed using the five-hole abdominal method (**Figure 3**). A 10-mm trocar was placed at the lower edge of the umbilical foramen as hole A. A 5-mm trocar was placed 1.0 cm below the xiphoid process as hole B. A 10-mm trocar was placed at the midpoint of the line connecting holes B and A, thus creating hole C. A 10-mm trocar was horizontally placed 3.0 cm above the left clavicle midline umbilicus as hole D. A 5-mm trocar was placed below the left axillary anterior rib as hole E. The lead surgeon used holes B and C during surgery; however, hole C was the main operating hole. The first assistant used holes D and E.

During surgery, inversion of the patient’s internal organs was confirmed, and cirrhotic changes in the liver were observed. No tumors were observed on the surface; however, a tumor was located in the deep part of the liver parenchyma.

**Figure 4:**
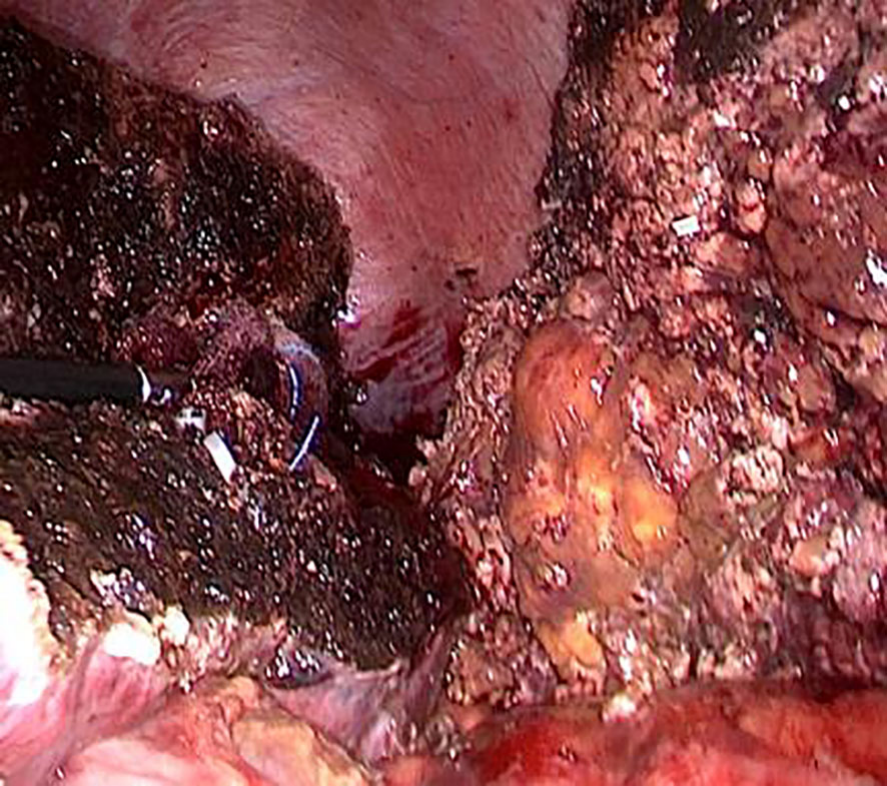
Complete removal of the tumor during surgery.

After releasing the perihepatic ligament, the lesser sac was opened, and a preblocking band was placed at the first hepatic hilum. A resection line was created by using the intraoperative ultrasound on the liver surface to denote the approximate range of the tumor in the right posterior lobe of the liver. Thereafter, the liver tumor was completely removed along that line using an ultrasonic knife (**Figure 4**).

**Figure 5:**
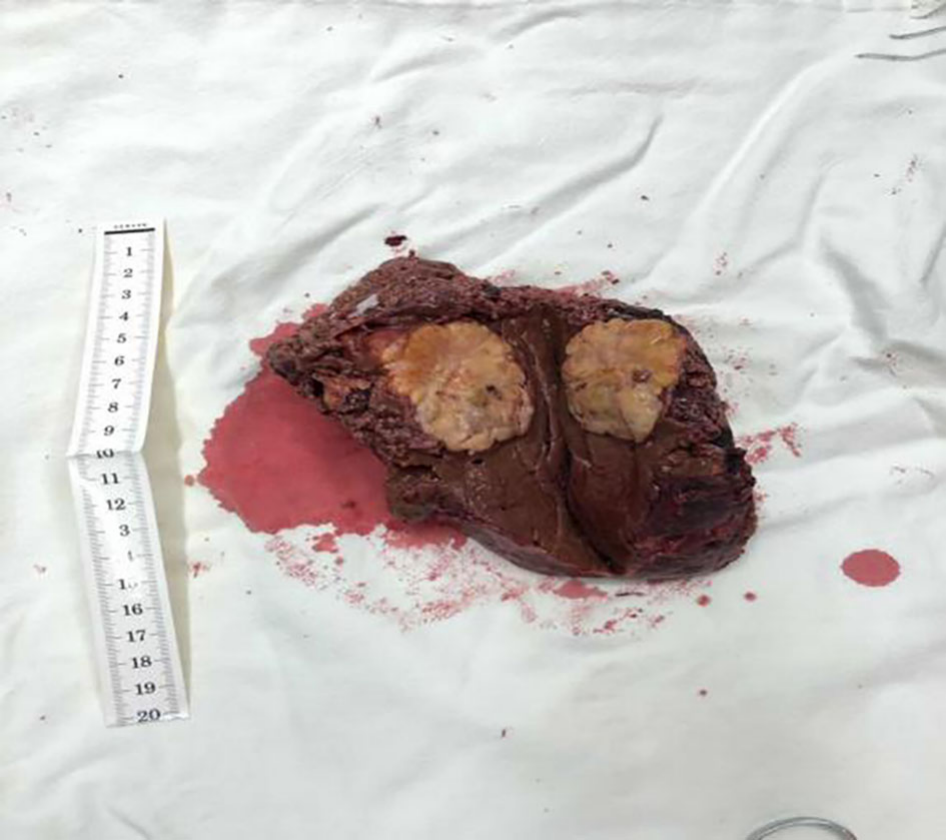
A tissue sample that was removed during surgery.

The pipeline structure was closed with a Hem-o-lock clamp (Teleflex Medical, Wayne, PA, USA) or silver clamp, and the active bleeding site was closed with Prolene sutures (Ethicon, Somerville, NJ, USA) to further improve wound hemostasis. Holes B (1.0 cm below the xiphoid process) and C (the midpoint of the line connecting holes B and A) were connected to create a midline incision with a length of 6.0 cm in the upper abdomen, and the specimen was removed. The wound was rinsed with warm distilled water, and the specimen was placed in a self-made collection bag. After checking the wound surface to ensure the absence of active bleeding and bile leakage, drainage pipes were placed under the diaphragm and liver and through holes D (3.0 cm above the left clavicle midline umbilicus) and E (below the left axillary anterior rib). During surgery, the first hepatic hilar space was blocked three times, resulting in a blood loss of approximately 1000 mL. Four units of suspended red blood cells and 400 mL of plasma were infused. The operative time was 208 min, and the postoperative anatomical specimens were intact (**Figure 5**).

**Figure 6:**
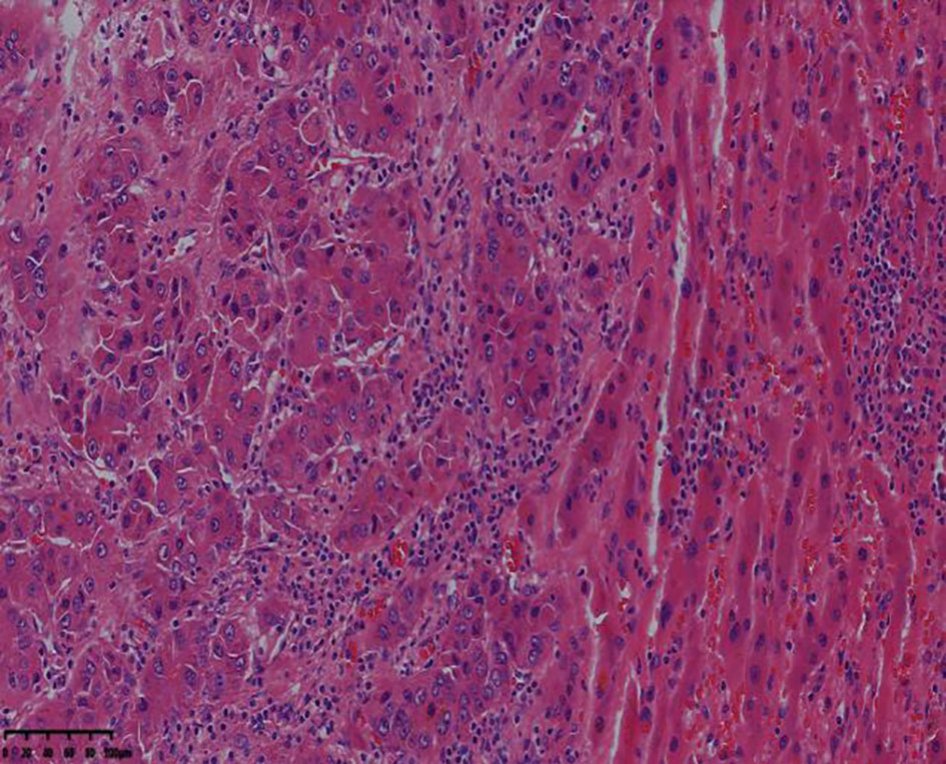
The resected lesion exhibits moderately differentiated hepatocellular carcinoma (hematoxylin-eosin staining, 4× magnification).

Complications such as bleeding, bile leakage, and infection were not observed during surgery, and the patient was discharged 8 days after surgery. The postoperative pathological examination indicated moderately differentiated hepatocellular carcinoma in the liver (5.6-cm × 5.0-cm × 4.5-cm gray-white and gray-yellow nodules), no clear intravascular tumor thrombus, a negative cutting edge, turbid and swollen surrounding liver cells, and chronic inflammatory cell infiltration in the portal area (**Figure 6**).

**Figure 7:**
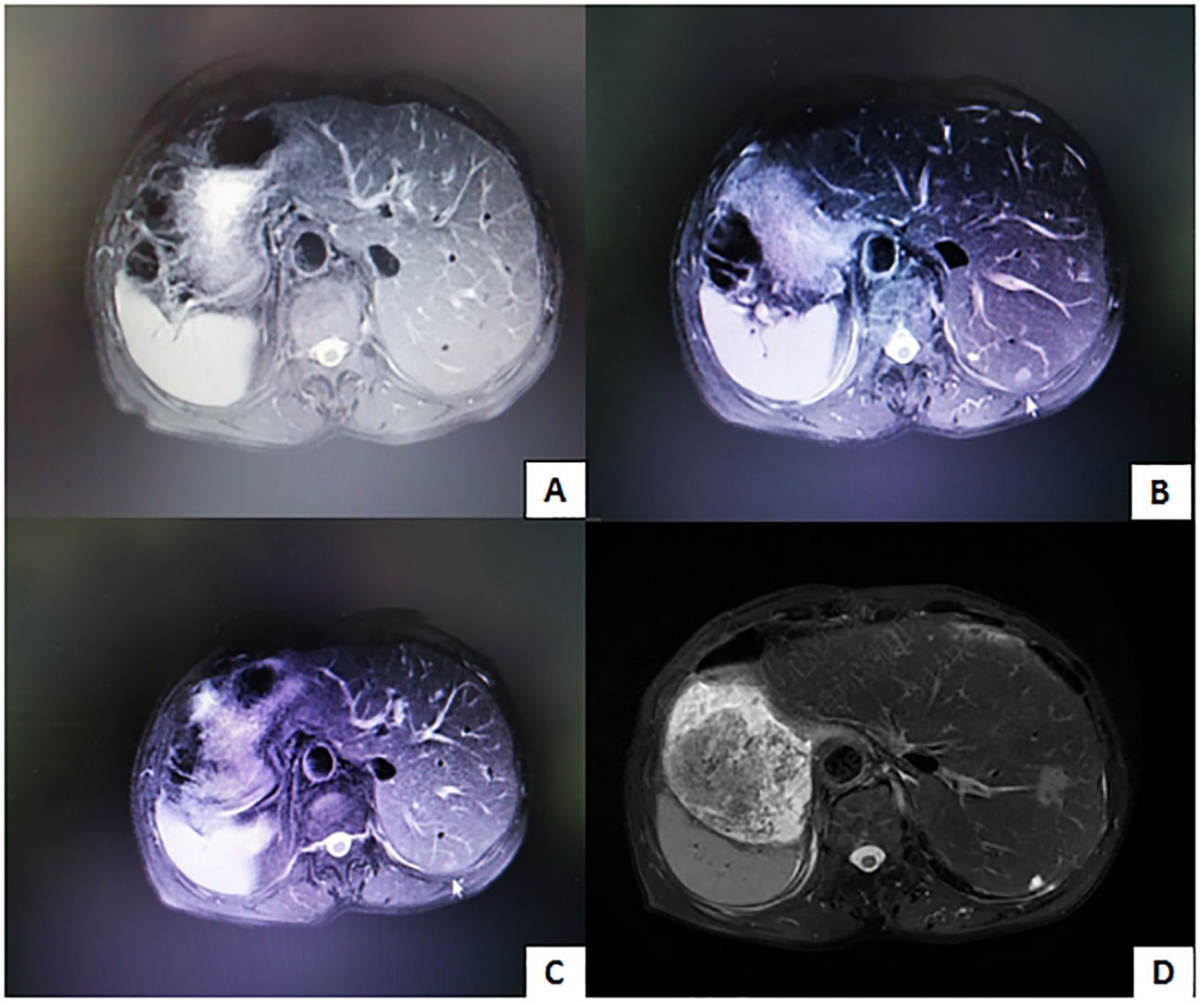
**(A)** Tumor recurrence is not observed 8 months after surgery. **(B)** Image obtained 13 months after surgery reveals new lesions. **(C)** Image obtained 15 months after surgery reveals thatthe lesion is smaller. **(D)** Image obtained 5 years after surgery reveals that the lesion is still small.

The patient underwent hepatic artery embolization three times after surgery, and tumor recurrence was not observed on abdominal MRI 8 months after surgery (**Figure 7A**). Because of the coronavirus disease 2019 (COVID-19) pandemic, the patient was not followed up for 5 months. Thirteen months after surgery, new tumors were found on abdominal MRI (**Figure 7B**), and hepatic artery embolization was performed twice. Fifteen months after surgery, MRI revealed that the tumor had shrunk (**Figure 7C**). The patient used oral antiviral therapy (tenofovir dipivoxil fumarate tablets 300 mg, twice per day) after surgery, and the alpha-fetoprotein and prothrombin levels were within the normal ranges. During approximately 5 years of follow-up, the patient’s condition was generally good and stable. Five years after surgery, MRI revealed that the lesion had remained small (**Figure 7D**).

## 3 Discussion and conclusions

The incidence of SIT is extremely low (5), and its etiology remains unclear. The possible mechanisms of its occurrence include rotational disorders during embryonic development and abnormalities in the chromosomes and genes of both parents. SIT is associated with the inheritance of an autosomal recessive gene (6, 7).

The principles of surgical treatment for patients with SIT and liver cancer are consistent with those for patients without SIT. However, variations in anatomy and changes in surgical habits can create surgical difficulty. The posterior position of the right lobe of the liver and thickened liver tissue made it difficult to expose the tumor and perform the surgery. Patients with SIT present unique surgical challenges during technically demanding laparoscopic resections due to their characteristic anatomic variations. The mirror-image orientation of vascular and biliary structures may lead to intraoperative disorientation, potentially compromising surgical precision. Notably, a thorough understanding of this reversed anatomy coupled with heightened intraoperative vigilance are critical determinants of procedural success.

Compared to surgery for patients with organs in their usual positions, surgery for those with SIT typically requires more time (8). Liver resection that involves complex vascular structures and SIT requires a highly skilled surgeon with a deep understanding of the anatomy. By reversing the positions of the lead surgeon and assistant, the surgery can be completed without complications.

However, during surgery, both the lead surgeon and the first assistant should use their forehand skills. Many laparoscopic surgeries have been successfully performed worldwide for patients with both SIT and cancer, including gastric (9) and lung (10) cancers. Given the rarity of SIT, current literature primarily consists of isolated case reports rather than systematic analyses with large sample sizes. Emerging evidence from these clinical observations suggests that laparoscopic procedures in SIT patients with malignancies do not appear to be associated with an increased risk of perioperative complications. Notably, both gastric and pulmonary carcinoma cases with SIT comorbidity have demonstrated favorable prognoses following minimally invasive laparoscopic interventions. Nevertheless, these preliminary findings require validation through multicenter studies with expanded cohort sizes to establish robust clinical evidence. However, only a few patients have both liver cancer and SIT. We successfully performed laparoscopic surgery in a patient with SIT and a concomitant tumor in the right posterior lobe of the liver.

SIT is a rare congenital anatomical anomaly associated with challenging surgical procedures and case management. With comprehensive preoperative examinations and meticulous intraoperative coordination, laparoscopic surgery is feasible in patients with SIT and liver cancer. Additional studies should be performed to explore the treatment and prognosis of these patients.

## Data Availability

The raw data supporting the conclusions of this article will be made available by the authors, without undue reservation.
